# Liver Xenotransplantation: From Early Primate Trials to the First-in-Human Porcine Bridging Therapies

**DOI:** 10.3390/jcm15083144

**Published:** 2026-04-20

**Authors:** Alexandru Grigorie Nastase, Alin Mihai Vasilescu, Ana Maria Trofin, Nicolae Florin Iftimie, Juan José Segura-Sampedro, Ramona Cadar, Iulian Buzincu, Alexandra Davidescu, Anda Lucia Nastase, Oana Georgiana Briceanu, Corina Lupascu-Ursulescu, Cristian Dumitru Lupascu

**Affiliations:** 1 Faculty of Medicine, Grigore T. Popa University of Medicine and Pharmacy, 700115 Iasi, Romania; alex.gr.nastase@gmail.com (A.G.N.); iftimienicolaeflorin@yahoo.com (N.F.I.); tibaramona@yahoo.com (R.C.); buzincu_iulian@yahoo.com (I.B.); alexandra.davidescu@umfiasi.ro (A.D.); anda-lucia.nastase@email.umfiasi.ro (A.L.N.); briceanugeorgianamedicina@gmail.com (O.G.B.); corina.ursulescu@gmail.com (C.L.-U.); cristian_lupascu@yahoo.com (C.D.L.); 2 General Surgery and Liver Transplant Clinic, St. Spiridon University Hospital, 700111 Iasi, Romania; 3 Peritoneal, Retroperitoneal and Soft Tissue Oncological Surgery Service, Department of General and Digestive Surgery, Hospital Universitario La Paz (IdlPaz), 28046 Madrid, Spain; 4 Faculty of Medicine, Universidad San Pablo CEU, 28040 Madrid, Spain; 5 Translational Research and Innovation Group in General and Digestive Surgery, Research Institute, Hospital Universitario La Paz (IdIPaz), 28046 Madrid, Spain; 6 Advanced Research and Development Center for Experimental Medicine “Prof. Ostin C. Mungiu”-CEMEX, Grigore T. Popa University of Medicine and Pharmacy, 700454 Iasi, Romania; 7 Radiology Clinic, St. Spiridon University Hospital, 700111 Iasi, Romania; 8 Academy of Romanian Scientists, 050044 Bucharest, Romania; 9 Romanian Academy of Medical Sciences, 030167 Bucharest, Romania

**Keywords:** liver xenotransplantation, porcine liver transplantation, genetically engineered pigs, acute liver failure, bridge-to-transplant therapy, hyperacute rejection, pig-to-human transplantation, auxiliary liver xenotransplantation, CRISPR/Cas9 genome editing, solid organ xenotransplantation

## Abstract

Liver transplantation remains the definitive treatment for end-stage liver disease and acute liver failure, yet a critical and persistent shortage of donor organs results in thousands of preventable deaths annually worldwide. Xenotransplantation has emerged as a potential solution to this structural deficit. This narrative review traces the evolution of liver xenotransplantation, from early non-human primate trials in the 1960s through the application of CRISPR/Cas9-driven multi-gene editing platforms in contemporary porcine donors. The immunological barriers that drove the transition from primate to porcine donors are examined, including hyperacute rejection mediated by anti-α-Gal antibodies, coagulation dysregulation and xenograft thrombotic microangiopathy. The genetic engineering strategies underlying current triple-knockout, ten-gene-edited donor pigs are reviewed alongside the preclinical non-human primate evidence establishing biological feasibility. The three pig-to-human liver xenotransplantation studies published between 2025 and 2026 are then analyzed, encompassing heterotopic auxiliary transplantation in a brain-dead decedent, extracorporeal liver cross-circulation and the first auxiliary liver xenotransplantation in a living recipient with a documented 171-day survival. These cases collectively provide preliminary evidence supporting proof-of-concept for porcine hepatic bridging therapy, with current evidence supporting a role for xenogeneic liver support as a temporary bridge to recovery or allotransplantation rather than definitive organ replacement. Xenograft thrombotic microangiopathy is identified as the principal remaining biological barrier, and the substantial translational challenges, including reproducibility, scalability and regulatory readiness that must be resolved before broader clinical application can be considered.

## 1. Introduction

Liver transplantation (LT) represents one of the most transformative achievements of twentieth-century medicine. Since Thomas Starzl performed the first human liver transplant at the University of Colorado in 1963 [1], the field has undergone a series of paradigm-shifting advances, from the introduction of calcineurin inhibitor immunosuppression in the 1970s and 1980s to the contemporary adoption of machine perfusion, such that one-year and five-year post-transplant survival now routinely exceed 90% and 85%, respectively [2]. Despite these advances, liver disease remains an enormous global burden responsible for approximately 2 million deaths annually worldwide, and LT, the definitive treatment for end-stage liver disease and acute liver failure (ALF), is limited in its reach by a persistent and structural shortage of donor organs [2,3,4].

The gap between organ supply and demand is not merely a logistical challenge but a structural ceiling inherent to reliance on human allografts. Despite decades of coordinated effort to expand the donor pool through donation after circulatory death programs, living donor surgery and improved organ preservation technology, this deficit has sustained scientific interest in xenotransplantation, the transplantation of organs from a non-human species, as a theoretically inexhaustible alternative organ source. Advances in CRISPR/Cas9-based genome engineering have transformed this aspiration into an active translational program, enabling the creation of multi-gene edited pigs whose organs increasingly approximate the immunological and physiological profile of their human counterparts, with genetically modified pig organ graft survival extending to years in non-human primate (NHP) models [5].

This is a narrative review conducted without a predefined systematic search protocol or formal inclusion criteria. A literature search was performed using PubMed, Scopus and Google Scholar, with no date restrictions, using terms including “liver xenotransplantation”, “porcine liver transplant”, “genetically engineered pig”, “xenograft rejection” and related combinations. In order to capture both the historical context and the most recent clinical advancements, these database searches were conducted in two phases: an initial search in August 2025, followed by an updated search in March 2026. Final source selection was based on relevance to the review objectives and the authors’ expert judgement. The aim of this review is to trace the evolution of liver xenotransplantation (LxT), from early experiments in NHP models to the landmark first-in-human porcine liver procedures reported between 2025 and 2026. It examines the genetic engineering strategies underpinning the current generation of donor pigs, underlining the preclinical evidence establishing biological feasibility and the clinical, immunological and ethical lessons derived from the three pig-to-human LxT procedures performed to date. Through this synthesis, the review aims to define both the current state of the field and the research priorities that will determine whether liver xenotransplantation becomes a durable and scalable component of the transplantation arsenal.

### 1.1. The Clinical Need for Alternative Liver Support

The scale of this unmet need is well documented across regions with highly developed transplant systems, underscoring that the deficit is structural rather than organizational. In the United States, more than 10,000 liver transplants were performed in 2023, a record figure. Yet, 9745 adult patients remained on the waiting list (WL) at year’s end and 943 died awaiting a suitable organ [6]. An estimated additional 35,000 deaths from chronic liver disease occur annually among patients who never reach the WL [7]. The situation is comparable in Europe: at the end of 2025, 1658 patients were actively listed across the Eurotransplant region (Austria, Belgium, Croatia, Germany, Hungary, Luxembourg, the Netherlands and Slovenia), where 18% of LT candidates die on the WL and a further 4% are delisted due to clinical deterioration [8,9]. Single-center data from Germany corroborate this, with 27.7% of liver WL candidates removed due to death or clinical deterioration [10].

**Figure 1:**
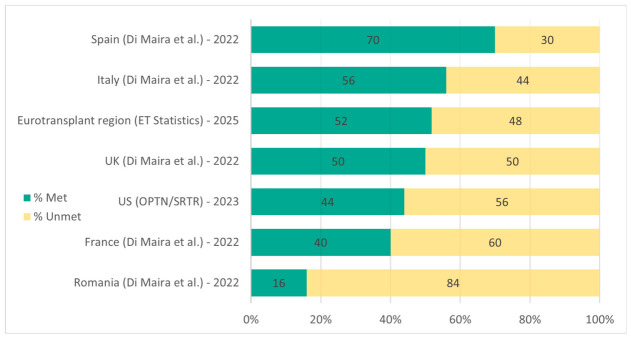
Liver transplants demand met versus unmet in selected countries and regions (2022–2025). Horizontal stacked bars show the proportion of liver transplant demand fulfilled (% Met, green) and remaining unmet (% Unmet, yellow). Data for Spain, Italy, UK, France and Romania are from Di Maira et al., 2025 [11]; US data from Kwong et al., 2025 [6]; Eurotransplant 2025 totals from Eurotransplant Statistics [8]. Bars are ordered from highest to lowest % demand met, highlighting regional differences in liver transplant coverage.

Country-level data from 2022 reflect the same pattern: transplantation rates covered approximately 40% of listed demand in France (1294 of 3219 active candidates), 56% in Italy (1479 of 2620), 50% in the United Kingdom (922 of 1836) and only 16% in Romania (75 of 467) [11]. Critically, even in countries with the highest donation rates worldwide, such as Spain, a substantial unmet need persists (Figure 1), reinforcing the conclusion that deceased and living human donors alone will never constitute a sufficient supply to meet transplant demand, establishing the rational basis for exploring xenotransplantation as a complementary strategy [12].

### 1.2. ALF and Bridging Therapy Principles

ALF is a rapidly progressive medical emergency characterized by hepatic dysfunction, including coagulopathy and encephalopathy, which may result in multiorgan system failure (MOSF) [13]. This rapid progression to MOSF leads to mortality rates of 80–90% in the absence of LT. An analysis of data collected by the ALF Study Group found that only 50% of ALF patients survive without LT, with survival depending on factors such as the coma scale at admission, vasopressor use, international normalized ratio (INR) and bilirubin levels [14].

In order to understand the design and necessity of bridging therapies (BTs), the complex pathophysiology of ALF needs to be addressed. The massive necrosis of hepatocytes releases damage-associated molecular patterns (DAMPs) into the circulation [15]. These molecules activate the innate immune system, triggering a cytokine storm characterized by elevated levels of tumor necrosis factor-alpha, interleukin-1 and interleukin-6 [16]. This systemic inflammation mimics sepsis and leads to vasodilation, hypotension and MOSF, particularly acute kidney injury and respiratory failure [17]. Simultaneously, the failing liver loses its capacity to clear neurotoxins, most notably ammonia, resulting in hyperammonemia that leads to astrocyte swelling, cerebral edema and intracranial hypertension [18]. Furthermore, the liver’s synthetic failure leads to a profound deficit in pro and anticoagulation factors, creating a fragile hemostatic balance that complicates invasive procedures [19].

Unfortunately, unlike the kidney, which can be supported with dialysis for more than 5 years [20], or the heart, which can be supported with ventricular assist devices for more than 4 years [21,22], there is currently no equivalent long-term support for the liver [23]. Modern management of ALF includes intensive care support (fluids, vasopressors, ventilation) and removal of toxins through blood purification [24]. In practice, combinations such as continuous renal replacement therapy (CRRT) plus plasma exchange or albumin dialysis are used to remove ammonia and inflammatory mediators, improving stability while awaiting LT [18]. Conventional non-bioartificial blood purification systems can reduce endotoxins and ammonia, thereby stabilizing the patient; however, they cannot fully restore hepatic biosynthesis, metabolism or detoxification and therefore cannot substitute the liver’s vital functions [25]. As a result, BTs that are able to provide complete hepatic function, even temporarily, are critically needed when organs are not available or the patient is not an immediate transplant candidate.

**Figure 2:**
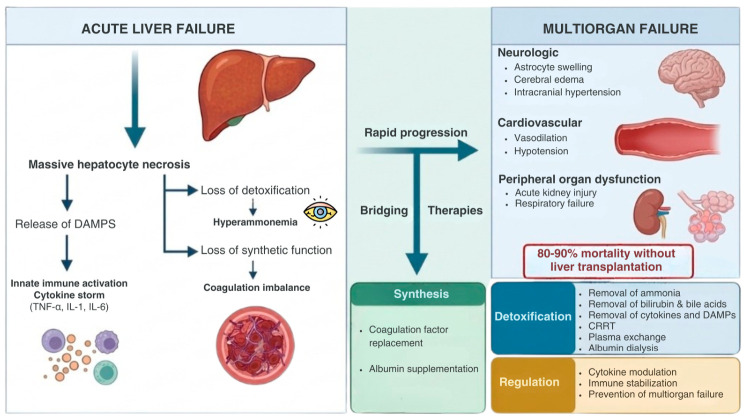
Pathophysiology of ALF, progression to multiorgan failure and therapeutic targets of bridging therapies.

The fundamental concept of BTs in ALF is dual: first, to act as a bridge to recovery by stabilizing liver function and allowing the native liver to recover; and second, to act as a bridge to transplantation, by maintaining the patient’s physiological viability until a suitable human donor organ becomes available [26]. BTs target three distinct physiological domains (Figure 2):

- Detoxification: The removal of water-soluble toxins (ammonia), albumin-bound toxins (bilirubin, bile acids) and inflammatory mediators (cytokines, DAMPs) [16,27];
- Synthesis: The replenishment of vital plasma proteins, such as coagulation factors and albumin [28];
- Regulation: The modulation of dysregulated immune response and systemic inflammation to prevent MOSF [27,29].

## 2. The Evolution of Liver Xenotransplantation: From Primates to Pigs

The history of LxT is defined by extraordinary human ambition, repeatedly confronted by the harsh biological realities of species incompatibility. Unlike the kidney or heart, the liver is a metabolic powerhouse with complex synthetic, secretory and immunological functions, making it a uniquely challenging organ to replace [30]. The early history of this field is dominated by the use of NHP, specifically chimpanzees and baboons, as donors. This era, spanning from the mid-1960s to the early 1990s, was driven by the phylogenetic proximity of these species to humans, which investigators hypothesized would mitigate the fierce immunological rejection [31].

### 2.1. The Pre-Cyclosporine Era: Chimpanzee Trials (1966–1974)

In the 1960s, treatment options for end-stage organ failure were extremely limited, as dialysis was rare and brain death was not yet legally defined, making human organs scarce. In this context, LxT arose not as a curiosity, but as a desperate last resort for patients facing imminent death [32].

In 1963, Keith Reemtsma performed a series of chimpanzee-to-human kidney transplants at Tulane University, with one patient surviving for nine months, showing that organs from closely related species could sustain human life and withstand hyperacute rejection with proper immunosuppression [33]. Thomas Starzl observed that, unlike rhesus monkey grafts, chimpanzee organs appeared to enjoy a degree of immunological privilege due to their genetic closeness to Homo sapiens [34].

Between 1966 and 1974, Starzl and his team performed three orthotopic chimpanzee-to-human LxT, in an era when immunosuppression was limited to azathioprine, corticosteroids and antilymphocyte globulin. The first, in a pediatric patient in 1966 and two subsequent procedures in 1969 and 1974, all yielded poor outcomes. Graft function was short-lived and the longest survivor lived only 14 days, with all recipients dying within two weeks. In parallel, Starzl explored the chimpanzee liver as an extracorporeal bridge in fulminant hepatic failure, demonstrating brief functional support, but no durable clinical benefit. By 1974, disappointing results, increased availability of human organs after the recognition of brain death and ethical concerns surrounding great apes ended this era. Despite its failure, the program established the lasting proof-of-concept that an NHP liver could function, though only temporarily, in the human circulation [35].

### 2.2. The FK506 Era: Baboon Clinical Trials (1992–1993)

The field of LxT remained largely dormant until the early 1990s, when worsening organ shortages and the clinical introduction of Tacrolimus (FK506) in 1989 reignited interest. For this renewed series of trials, the donor species was shifted from chimpanzees to baboons, which were more ethically acceptable and available from breeding colonies [35].

The first patient selected for the historic baboon-to-human LxT was a 35-year-old male with end-stage cirrhosis secondary to chronic hepatitis B virus (HBV) infection, coinfected with HIV, who was excluded from the human LT WL. On June 28, 1992, he underwent orthotopic LxT using a 15-year-old male baboon liver weighing approximately 600 g. To accommodate the size mismatch and preserve hemodynamic stability, the surgical team employed a piggyback technique. The patient was extubated and was reportedly eating and walking within five days. Immunosuppression consisted of FK506, prednisone, prostaglandin and cyclophosphamide. The graft functioned immediately, producing bile and clearing toxins, serial biopsies revealing minimal cellular rejection. Over time, the patient’s serum proteins, including albumin and clotting factors, shifted from human to baboon isoforms, demonstrating that the xenograft was actively synthesizing functional proteins sufficient to support human physiology, effectively rendering the patient a chimera. Despite this, the patient developed renal failure after the first month, metabolic disturbances and a rapid graft enlargement to 1555 g, reflecting the liver’s adaptive response to human metabolic demands. He died 70 days post-transplant, the terminal event being subarachnoid cerebral hemorrhage caused by invasive aspergillosis. The autopsy revealed a viable liver with preserved architecture and minimal rejection, but with a biliary tree filled with biliary sludge, indicating the physiological incompatibility between the baboon liver and the human biliary system [36].

Encouraged by the 70-day survival and the resistance of the baboon liver to the HBV, Starzl performed a second LxT in January 1993. The recipient was a 62-year-old male, also suffering from HBV-induced cirrhosis. The protocol was similar, utilizing a baboon donor and the FK506 regimen. The patient developed peritonitis secondary to an anastomotic leak at the biliary reconstruction site, resulting in his death at 26 days post-transplant [37,38].

### 2.3. Analysis of the Primate Era Failures

The autopsy reports and retrospective analyses of Starzl’s baboon clinical trials identified the immunological, physiological and ethical barriers that ultimately drove the transition to the porcine models:

- The Limit of Cellular Immunosuppression: While FK506 successfully suppressed T-cell mediated rejection, it could not control the innate immune response. Analyses of these cases suggested that uncontrolled complement activation contributed to graft damage, with the regulatory proteins being mismatched across species. Complement pathways generate C3 and C5 fragments and the membrane attack complex, which is associated with endothelial activation and innate immune injury that cannot be controlled by cellular immunosuppressants alone. These findings highlight the role of antibody and complement-mediated humoral xenograft injury as a significant immunological barrier even between phylogenetically close species, further suggesting that one approach to overcome this barrier could be the use of transgenic donor animals expressing complement regulatory proteins to protect the xenograft from antibody-mediated injury [36,39,40,41].
- Physiological Incompatibility: The baboon LxT demonstrated that immunological acceptance does not guarantee physiological compatibility. In the first clinical case, widespread biliary sludge occupied the entire intrahepatic biliary tree, despite a patent choledochojejunostomy, with the necropsy revealing the fatal downstream consequences of bile stasis and sludge formation. These findings highlight the subtle but crucial interspecies differences in hepatobiliary physiology that contributed to graft dysfunction [36,42].
- The Zoonosis and Ethical Barrier: The use of baboons raised significant concerns about the transmission of novel retroviruses (simian foamy virus, simian immunodeficiency virus) into the human population [43]. Unlike pigs, which have lived alongside humans for thousands of years, primates carry viruses that are evolutionarily ready to jump to humans [44]. Combined with the ethical concerns surrounding the use of intelligent primates as “spare parts”, the risk–benefit ratio became difficult to justify given the limited survival [45,46,47].

**Figure 3:**
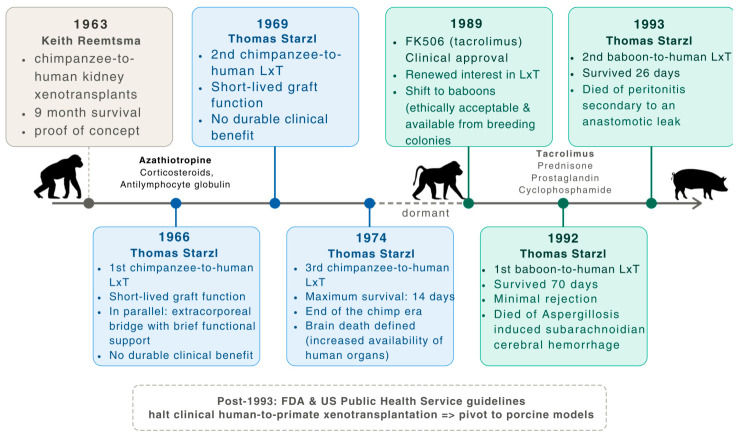
Timeline of the major milestones in LxT, illustrating the progression from primate donors to the shift toward porcine models.

Consequently, growing concerns about public health and cross-species infection led the FDA and the U.S. Public Health Service to issue guidelines effectively halting clinical primate xenotransplantation [48]. The field pivoted decisively toward pigs, a species that offered a virtually unlimited supply while presenting different and formidable immunological challenges [49]. This timeline illustrating the evolution of LxT from primates to pigs is summarized in Figure 3:

## 3. The Shift to Pigs and the Rise of Genetic Engineering

The shift from primate to pig donors in the late 1990s represented more than a simple change of species. It marked a fundamental rethinking of the approach to xenotransplantation. Throughout much of the 20th century, research focused on non-human primates because their close evolutionary relationship to humans suggested that conventional immunosuppression might suffice to prevent graft rejection [46]. In contrast, the adoption of pigs acknowledged that the donor organs would be inherently immunologically incompatible, necessitating genetic engineering to render them suitable for human recipients [50]. This shift was necessitated by the logistical impossibility of farming primates for organs and the ethical or safety profile of the pig (Sus scrofa domesticus), which is physiologically similar to humans in organ size and function, breeds rapidly and is already utilized for food [46,51].

### 3.1. The Immunological Barrier: Hyperacute Rejection

In early pig-to-primate xenotransplantation experiments, the most immediate barrier was hyperacute rejection (HAR), a rapid immune response triggered when preformed natural antibodies in the recipient recognize porcine antigens and activate complement [52]. Pigs express a functional gene encoding α-1,3-galactosyltransferase (GGTA1), which synthesizes the α-Gal epitope on glycoproteins and glycolipids throughout porcine tissues, especially on vascular endothelium [53,54]. Humans, apes and Old World monkeys, in contrast, lack a functional GGTA1 gene and therefore do not express α-Gal [55]. Evolutionary inactivation of GGTA1, millions of years ago, in these primates led to the development of circulating IgM and IgG antibodies against α-Gal, likely induced by lifelong exposure to gut microbes carrying structurally similar sugars [56,57]. These anti-α-Gal antibodies, which can constitute up to 1–4% of total immunoglobulins in humans, bind within seconds to α-Gal epitopes on porcine endothelial cells during graft perfusion [58]. Antibody binding to porcine endothelial antigens initiates activation of the classical complement pathway, resulting in complement-mediated endothelial activation and injury. Complement activation promotes loss of endothelial barrier function and conversion of the endothelium to a procoagulant state, with subsequent vascular leakage, interstitial edema, hemorrhage and intravascular thrombosis that progressively compromise graft perfusion [59]. In wild-type pig-to-primate models, this complement-mediated injury historically destroys the graft within minutes to hours [52].

### 3.2. The Genetic Revolution: Rewriting the Porcine Genome

During the 1990s and early 2000s, before gene-editing technologies like knockout pigs became widely available, researchers focused on protecting the pig endothelium from complement-mediated damage as a strategy to prevent HAR in xenotransplantation [60]. To achieve this, scientists created transgenic pigs that expressed human complement regulatory proteins on their cell surfaces [61]. The most common were human decay-accelerating factor (hDAF/CD55), membrane cofactor protein (hMCP/CD46) and membrane inhibitor of reactive lysis (hMIRL/CD59) [62]. These proteins inhibit the complement cascade at different points: CD46 acts as a cofactor for inactivating the C3 convertase complex, CD55 accelerates the decay of complement convertases and CD59 blocks formation of the membrane attack complex [63]. Organs from these transgenic pigs could resist the immediate destructive effects of human antibodies, effectively preventing HAR. However, even with this protection, a slower form of antibody-mediated injury, known as acute humoral xenograft rejection, still occurred, highlighting the need for additional strategies to control xenograft immune responses [64].

A decisive breakthrough in xenotransplantation occurred in 2002–2003 with the generation of pigs lacking GGTA1, commonly referred to as α-1,3-galactosyltransferase gene-knockout (GTKO) pigs [64]. By genetically deleting GGTA1, researchers produced pigs that no longer expressed the α-Gal carbohydrate antigen on their cells [65]. Because α-Gal is the dominant target of naturally occurring human anti-pig antibodies, its removal dramatically reduced antibody binding and effectively eliminated classical HAR [66]. While GTKO pigs addressed the major barrier posed by α-Gal, subsequent studies revealed that humans also possess antibodies directed against additional porcine carbohydrate antigens, notably N-glycolylneuraminic acid (Neu5Gc) synthesized by the cytidine monophospho-N-acetylneuraminic acid hydroxylase (CMAH) gene and the DBA-reactive glycans (Sd(a)) antigen, controlled by β-1,4-N-acetylgalactosaminyl transferase 2 (β4GalNT2) [67]. Recognition of these residual antibody targets led to the development of pigs with combined deletions of GGTA1, CMAH and β4GalNT2, the so-called triple-knockout (TKO) pigs. These animals lack all three major carbohydrate xenoantigens and show markedly reduced binding of human IgM and IgG, representing a critical advance toward minimizing antibody-mediated xenograft injury [67,68].

### 3.3. The Transgenic Era—Current State

**Figure 4:**
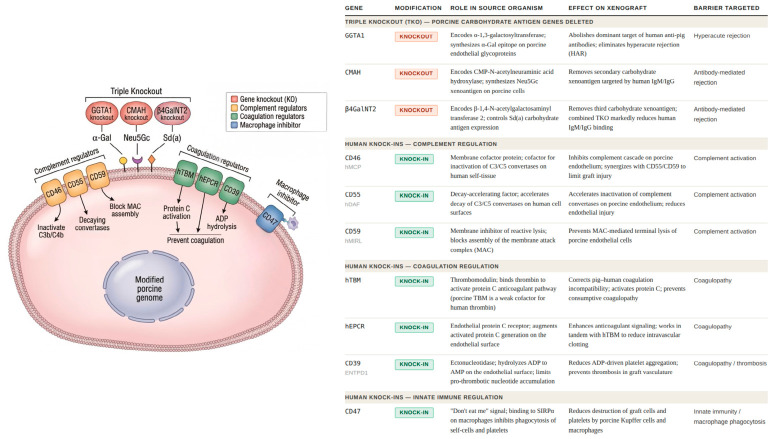
Genetic modifications in current-generation donor pigs for xenotransplantation. (**Left**) Schematic representation of a genetically modified porcine cell expressing the 10-gene modification panel. Three porcine carbohydrate antigen genes: GGTA1, CMAH and β4GalNT2, are knocked out, abolishing the α-Gal, Neu5Gc and Sd(a) xenoantigens respectively. Seven human transgenes are knocked in: complement regulators CD46, CD55 and CD59; coagulation regulators hTBM, hEPCR, CD39 and CD47, a macrophage inhibitor. (**Right**) Summary table of all ten modifications, grouped by the immunological or physiological barrier targeted: hyperacute rejection, antibody-mediated rejection, complement activation, coagulopathy and innate immune-mediated injury.

In the current state of xenotransplantation, donor pigs are engineered with an array of human genes on top of key knockouts to overcome both immunological and physiological barriers. Modern donor pigs carry the TKO of pig carbohydrate antigens (genes GGTA1, CMAH, β4GalNT2), plus seven human knock-in genes. These transgenes include human complement regulators (CD46, CD55, CD59); coagulation regulators: human thrombomodulin (hTBM); endothelial protein C receptor (hEPCR), CD39; and a macrophage inhibitor (CD47). Together, these modifications are designed not only to prevent immune rejection, but to correct the coagulation and inflammatory mismatches that affected earlier pig-to-primate liver grafts. For example, adding hTBM and hEPCR enhances the protein C anticoagulant pathway, while human CD39 degrades pro-thrombotic nucleotides, all aimed at preventing the rapid consumptive coagulopathy seen in pig livers. Likewise, human CD47 provides a “don’t eat me” signal to macrophages, reducing phagocytosis of graft cells and platelets [69,70]. These features are summarized below (Figure 4):

- Triple-Knockout of Carbohydrate Xenoantigens: The pig’s GGTA1, CMAH and β4GalNT2 genes are knocked out to eliminate α-Gal, Neu5Gc and Sd(a) antigens. This abolishes the major targets of human natural antibodies that trigger hyperacute/antibody-mediated rejection. In practice, 10-gene pigs show virtually minimal rejection and diminished early complement activation [67,71,72].
- Human complement regulators (CD46, CD55, CD59): All are expressed on pig endothelial cells to inhibit the human complement cascade. CD46 serves as a cofactor to inactivate C3/C5 convertases, CD55 accelerates decay of C3/C5 convertases and CD59 blocks the membrane attack complex [62,66,73]. These transgenes together “curb antibody-mediated rejection” by preventing excessive complement activation on the graft. In fact, the 10-gene pig liver graft showed minimal C4d deposition and no hyperacute rejection in vivo, validating this protection [69].
- Human Coagulation Regulators (hTBM, hEPCR, CD39): These transgenes counter pig–human clotting incompatibilities [65]. hTBM binds thrombin to activate protein C (anticoagulant) and human EPCR augments activated protein C generation; CD39 (ENTPD1) hydrolyzes ADP to AMP to prevent platelet aggregation [58,74]. Together, they are intended to prevent fatal consumptive coagulopathy (intravascular clotting) that otherwise occurs in pig liver grafts [58]. By contrast, pig thrombomodulin is a weak cofactor for human thrombin, so hTBM is critical for anticoagulation compatibility [75]. These modifications, together with additional therapeutic strategies, have significantly prolonged graft survival, allowing pig livers in certain primate models to function for several weeks instead of the few days reported in earlier studies [38].
- Human CD47 (“Don’t Eat Me” Signal): Overexpressed human CD47 on pig cells binds to SIRPα on human macrophages, sending a signal to inhibit phagocytosis. In pig-to-primate liver models, this aims to reduce the rapid destruction of human platelets and possibly graft cells by pig Kupffer cells. The 10-gene combination tackles the immune and physiological hurdles identified in earlier (primate-derived) liver xenografts: it prevents the antibody/complement attack while also aligning coagulation and macrophage interactions more closely to human physiology [69,70].

The pig-to-NHP model has served as the rigorous proving ground for these genetic advancements [38]. Because Old World monkeys (baboons and rhesus macaques) share the anti-Gal antibody profile with humans, they are the most relevant model for testing safety and efficacy before human trials [46,76]. The history of these experiments is a chronological march through increasingly sophisticated genetic modifications, with each era revealing a new biological hurdle.

The first pig-to-primate LxT were performed by Sir Roy Calne in Cambridge in 1968. Using wild-type pigs and baboons, Calne’s team attempted orthotopic LxT [77,78]. The results were immediate and devastating. The livers, unprotected from the baboon’s anti-Gal antibodies, underwent HAR. However, unlike kidneys, which simply ceased blood flow, the livers sequestered massive amounts of blood. The recipient animals developed profound thrombocytopenia and coagulopathy, dying from uncontrollable hemorrhage within 6 to 30 h, with the longest survivor in this early series living only 3.5 days [79,80,81].

Attempts in the 1990s using immunosuppression failed to improve these results significantly. The barrier of HAR was absolute: as long as the α-Gal antigen was present, the complement system would destroy the graft vasculature and the coagulation system would consume the recipient’s platelets [79,82].

The first significant extension of survival came with the introduction of pigs transgenic for hDAF(CD55). In 2000, Ramirez et al. transplanted livers from hCD55-pigs into baboons. The expression of hDAF successfully inhibited the complement cascade, preventing the immediate HAR. For the first time, pig livers functioned for days rather than hours. The longest surviving baboon lived for 8 days, maintaining metabolic function [83]. However, the animals eventually died from a combination of delayed humoral rejection and, more critically, a profound coagulopathy [81,82,83]. This era highlighted a new, non-immunological barrier: thrombocytopenia [84]. Even when the complement was blocked, baboon platelet counts dropped shortly after reperfusion [85]. This was not due to antibody binding but to the inherent incompatibility between porcine endothelial receptors and primate platelets [86,87].

Research into the hematologic and coagulation failures of LxT has identified the following distinct molecular mechanisms driving thrombocytopenia and coagulation dysregulation in the recipient:

1. von Willebrand Factor (vWF) Mismatch: Porcine vWF is a large multimeric glycoprotein that binds aberrantly with primate platelet glycoprotein Ibα. Unlike human vWF, porcine vWF can bind and activate primate platelets without the normal requirements for high shear stress, promoting platelet aggregation and contributing to platelet consumption and thrombocytopenia in pig-to-primate LxT models [88,89,90].
2. Macrophage Phagocytosis: Porcine Kupffer cells express SIRPα receptors that exhibit functional incompatibility with the CD47 “don’t eat me” signal on primate platelets, leading to impaired inhibitory signaling and permitting increased phagocytosis of the recipient’s circulating platelets [90,91].
3. Asialoglycoprotein Receptor 1 Sequestration: This porcine receptor, predominantly expressed on liver sinusoidal endothelial cells, binds to exposed galactose and N-acetylglucosamine residues on primate platelets, mediating their rapid phagocytic clearance from the circulation [92,93].
4. Thrombotic Microangiopathy: Porcine thrombomodulin exhibits a functional interspecies incompatibility, failing to adequately activate primate Protein C. This critical loss of anticoagulant regulation leaves the recipient in a profound procoagulant state, resulting in widespread fibrin deposition within the graft microvasculature (xenograft thrombotic microangiopathy—xTMA) and subsequent ischemic necrosis [90,94,95].

To address these defects, recent preclinical successes have relied on a combination of baseline genetic engineering and aggressive pharmacological support. In a pivotal 2017 study using GTKO pigs, Shah et al. recognized that the xenograft could not synthesize compatible clotting factors. To counter this, they supplemented the baboon recipients with continuous infusions of human Prothrombin Complex Concentrate. Furthermore, they augmented the baseline immunosuppressive regimen (which included FK506) with an anti-CD40 costimulation blockade, suppressing antibody production more effectively than calcineurin inhibitors alone. The results were unprecedented: survival extended to 25 and 29 days. The liver grafts maintained normal histology and synthetic function for nearly a month. While platelet counts remained low, the coagulopathy was managed well enough to prevent lethal bleeding for weeks. This established a new paradigm in NHP models, proving that prolonged survival is achievable if coagulation is aggressively managed. Ultimately, it paved the way for modern multi-gene (10-gene) pigs, which are engineered to do intrinsically what this study achieved pharmacologically [96].

## 4. The Translational Shift: The First Pig-to-Human Liver Xenotransplantations as Bridging Therapies (2025–2026)

The transition from NHP models to human recipients marks the most significant milestone in the history of LxT. While early preclinical research established the fundamental immunological barriers of cross-species grafting, the clinical application of these findings remained entirely theoretical until the emergence of highly sophisticated, multiplexed gene-editing platforms utilizing CRISPR/Cas9 for targeted knockouts and PiggyBac transposon systems for multiplex human transgene knock-ins [66,97]. Between 2025 and 2026, three landmark studies provided preliminary evidence that genetically modified porcine livers may be integrated into human circulatory systems, offering critical metabolic, synthetic and detoxification functions. These studies, ranging from extracorporeal perfusion in human decedents to orthotopic auxiliary implantation in a living patient, provide a profound window into the physiological compatibility, immune tolerance and unique pathophysiological challenges of pig-to-human LxT [98,99,100].

### 4.1. Heterotopic Auxiliary Transplantation in a Brain-Dead Recipient: The Tao et al. Study (2025) [98]

In a pioneering proof-of-concept study, Tao et al. evaluated the feasibility of heterotopic auxiliary LxT utilizing a brain-dead human recipient. The primary objective of this investigation was to observe short-term graft functionality, recipient hemodynamics and early xenogeneic immune responses over a predetermined ten-day monitoring period, simulating the temporal requirements of an acute bridging therapy [98].

#### 4.1.1. Donor Genetic Engineering and Recipient Preparation

To provide the xenograft, the researchers utilized a Bama miniature pig engineered with a highly specific 6-gene modification panel. Relying on the genetic principles established in earlier preclinical models, this graft featured the foundational TKO of carbohydrate xenoantigens: GGTA1, β4GalNT2, CMAH, alongside the insertion of three human transgenes targeting the complement and coagulation cascades: CD46, CD55, hTBM. To further mitigate early immunological risks, the brain-dead recipient was pre-screened to ensure low baseline levels of xenoreactive IgM and IgG, creating an optimal environment to evaluate graft functionality [98].

#### 4.1.2. Surgical Methodology and Anatomical Configuration

To avoid the physiological trauma and hemodynamic instability of a total native hepatectomy, the surgical team utilized a heterotopic auxiliary approach, leaving the recipient’s original liver in situ. After ensuring appropriate vessel caliber matching, the recipient’s infrarenal inferior vena cava (IVC) was partially resected. The graft’s suprahepatic IVC and portal vein were anastomosed to the proximal and distal ends of the transected IVC, respectively. Arterial inflow was established by bridging the porcine hepatic artery directly to the recipient’s abdominal aorta. Finally, to permit continuous quantification and biochemical analysis of hepatic output, the biliary drainage was externalized rather than anastomosed to the recipient’s enteral tract [98].

#### 4.1.3. Clinical Outcomes, Hemodynamics and Histological Evaluation

The xenograft demonstrated immediate and sustained functional viability. Goldish bile flowed from the external drain just two hours post-reperfusion, reaching a cumulative volume of 66.5 mL by postoperative day 10. Systemic synthetic function was further corroborated by progressively rising porcine liver-derived albumin in the recipient’s serum. Hepatic enzyme trends revealed a notable physiological divergence: while alanine aminotransferase (ALT) remained strictly within normal limits, aspartate aminotransferase (AST) spiked on postoperative day 1 before rapidly declining. This transient AST elevation, unaccompanied by ALT derangement, suggests an etiology related to initial ischemia–reperfusion injury or myocardial enzyme release, rather than intrinsic xenogeneic hepatocellular necrosis. Furthermore, late-stage elevations in bilirubin and γ-glutamyl transpeptidase were histologically attributed to mild intrahepatic cholestasis within the recipient’s native liver, rather than porcine graft failure [98].

Hemodynamic ultrasound monitoring confirmed excellent vascular patency. The porcine hepatic artery maintained high-velocity flow (41.45–60.63 cm/s) alongside stable portal and hepatic venous flow. Crucially, while early transient thrombocytopenia and prolonged activated partial thromboplastin time (aPTT) were observed, both parameters spontaneously recovered to baseline. This autonomous hematological recovery may suggest that the hTBM transgene contributed to mitigating the xenogeneic consumptive coagulopathy that historically plagued preclinical pig-to-primate models [98].

Histological and ultrastructural analyses on day 10 revealed no acute humoral or cellular rejection. The graft exhibited robust regenerative capacity, characterized by high hepatocyte proliferation (intense Ki67 positivity), extensive liver sinusoidal endothelial cell (LSEC) preservation (CD31 positivity) and minimal α-smooth muscle actin expression, indicating an absence of pathological stellate cell activation. Scanning electron microscopy (SEM) confirmed an intact microcirculation, revealing well-differentiated LSEC fenestrae free from microvascular thrombosis. Furthermore, rigorous viral surveillance revealed the absence of porcine endogenous retrovirus (PERV) transmission and porcine cytomegalovirus (PCMV) activity, supporting the safety of the designated pathogen-free donor breeding protocols [98].

### 4.2. Extracorporeal Liver Cross-Circulation (ELC) in Human Decedents: The Shaked et al. Study (2026) [99]

While auxiliary implantation represents an important proof-of-concept advance, it inherently requires an invasive laparotomy that imposes severe physiological stress. Seeking a less traumatic, rapidly reversible alternative for patients presenting with fulminant ALF, Shaked et al. explored the paradigm of ELC. Utilizing a four-subject human decedent model, this study evaluated the capacity of ex vivo, genetically modified pig livers to provide multi-day metabolic and synthetic support through a minimally invasive extracorporeal circuit [99].

#### 4.2.1. The Donor Profile and Circuit Architecture

The researchers utilized Yucatan miniature pigs, designated EGEN-5784, featuring a genetic modification profile designed to neutralize immunological recognition and coagulation discordance. Building upon a foundational TKO (GGTA1, CMAH, β4GalNT2), the EGEN-5784 donors expressed seven human transgenes hemizygously: hEPCR, hTBM, tumor necrosis factor alpha-induced protein 3 (TNFAIP3—acting as an anti-inflammatory regulator), Heme Oxygenase 1 (HMOX1), CD46, CD55 and CD47 (to inhibit macrophage phagocytosis). Finally, to mitigate zoonotic transmission risks, the pol gene of PERV was functionally inactivated [99].

The ELC system routed the decedents’ systemic venous blood to perfuse the ex vivo xenograft via a modified OrganOx metra circuit, an FDA-approved normothermic machine perfusion device. Executing a progressive study design, the researchers maintained the first three decedents on ELC for 72 to 84 h with their native livers left in situ. This initial cohort served to establish baseline feasibility, validating hemodynamic safety, physiological vascular flow dynamics and early immune tolerance [99].

#### 4.2.2. Anhepatic Stress Testing and Metabolic Efficacy

The critical metabolic stress test was conducted on Decedent 4. After an initial ELC phase, the surgical team performed a complete native hepatectomy with a portocaval anastomosis, rendering the decedent entirely anhepatic. The recipient was subsequently supported exclusively by the extracorporeal porcine liver for an unbroken period of 48 h [99].

During the anhepatic phase in Decedent 4, the ELC system achieved remarkable physiological stabilization. The acute acidosis that had developed intraoperatively during the hepatectomy procedure was rapidly and completely reversed upon the re-initiation of xenograft cross-circulation. Over the 48 h of exclusive extracorporeal support, the xenograft maintained stable metabolic parameters, including a normal physiological pH of 7.35, low systemic lactate concentrations of 2.3 mmol/L, normal ammonia clearance at 50 µmol/L and a perfectly stable coagulation profile with an INR of 1.1. The absolute, life-sustaining reliance of the human physiology on the xenograft was evident upon the elective disconnection of the ELC circuit. Following the cessation of extracorporeal support, the anhepatic decedent experienced rapid and catastrophic systemic collapse, developing severe vasoplegia requiring massive norepinephrine infusion, anuria, overwhelming lactic acidosis reaching 16 mmol/L and fatal hyperammonemia peaking at 89 µmol/L within hours, culminating in complete circulatory cessation [99].

Immunologically, the ELC model proved highly resilient under minimal baseline pharmacological immunosuppression, which primarily consisted of intravenous methylprednisolone, though specific decedents received supplemental intravenous immunoglobulin or pegcetacoplan to evaluate platelet mitigation. Post-perfusion histological analyses of the xenografts displayed well-preserved global parenchymal architecture. Immunohistochemistry revealed an inflammatory infiltrate composed predominantly of CD14+ macrophages and monocytes, with only a mild scattering of CD3+ T-cells localized primarily within the portal tracts. Although focal venule endotheliitis and mild red blood cell extravasation into the septal areas were noted by the 72 h mark, catastrophic HAR was entirely evaded and extensive microvascular thrombosis did not occur. This suggests a degree of efficacy of the EGEN-5784 genetic modifications in maintaining endothelial integrity within a human blood-contact model [99].

### 4.3. The First Auxiliary Liver Xenotransplantation in a Living Human: The Zhang et al. Study (2026) [100]

The culmination of preclinical primate testing and human decedent trials was realized by Zhang et al., who reported the world’s first successful auxiliary xenotransplantation of a 10-gene-edited porcine liver into a living human recipient. This landmark procedure represents an important step in transitioning LxT from a purely experimental exercise toward a potentially life-prolonging clinical intervention, though its compassionate use, single-patient context necessitates cautious interpretation [100].

#### 4.3.1. Clinical Rationale and Recipient Profile

The recipient was a 71-year-old male afflicted with hepatitis B-associated cirrhosis complicated by a massive, right-lobe hepatocellular carcinoma (HCC) measuring approximately 15 × 11 × 10 cm^3^. Comprehensive preoperative assessments revealed an alpha-fetoprotein level exceeding 100,000 ng/mL, an indocyanine green retention rate at 15 min of 16.5% and a severely diminished remnant liver-to-standard liver volume ratio of only 41.79%. Due to these profound functional impairments, the patient was deemed entirely ineligible for human allotransplantation under existing allocation criteria and native curative resection was contraindicated due to the near certainty of inducing lethal small-for-size syndrome and ALF. Facing imminent death from tumor rupture following the failure of transarterial chemoembolization, an auxiliary orthotopic LxT was authorized under compassionate use protocols as a desperate, life-saving bridging measure [100].

#### 4.3.2. 10-Gene Engineering and Surgical Implantation

The donor organ was procured from an 11-month-old Diannan miniature pig weighing 32 kg, specifically selected for its precise anatomical volumetric match with the adult human recipient. The liver was extensively engineered utilizing a 10-gene editing platform driven by CRISPR/Cas9 and PiggyBac transposon systems. This comprehensive array included TKO (GGTA1, CMAH, β4GalNT2) to prevent immediate humoral attack, three human complement regulatory transgenes (CD46, CD55, CD59), three human coagulation regulatory transgenes (CD39, hTBM, hEPCR) to enforce local antithrombotic activity and the critical macrophage-inhibitory signal CD47 to prevent phagocytic destruction of human platelets and erythrocytes [100].

Surgically, the massive right-lobe tumor was resected to eliminate the rupture threat and the porcine graft was implanted orthotopically into the newly vacated right hepatic fossa. The surgical team established vascular continuity utilizing native hepatic artery, portal vein and hepatic vein anastomoses, ensuring physiological inflow and outflow, while intentionally preserving the recipient’s functionally compromised left native lobe to serve alongside the xenograft. To safeguard against rejection, a rigorous, multi-agent immunosuppressive protocol was implemented. Induction therapy utilized rituximab for profound B-cell depletion, basiliximab for interleukin-2 receptor blockade and methylprednisolone. Maintenance therapy relied upon a carefully titrated combination of corticosteroids, FK506, sirolimus and mycophenolate mofetil (MMF), accompanied by periodic pulse dosing of basiliximab and rituximab [100].

#### 4.3.3. 171-Day Survival and Early Functional Milestones

The procedure was associated with an overall patient survival of 171 days. During the initial 31 postoperative days, the xenograft demonstrated effective metabolic function, producing consistent volumes of golden-yellow bile and synthesizing functional porcine albumin. The graft also secreted substantial levels of porcine coagulation factors, most notably porcine Factor VIII, with circulating concentrations rising to as much as 340-fold above the recipient’s baseline. This marked interspecies disparity significantly shortened the aPTT and led to the development of an early hypercoagulable state requiring therapeutic anticoagulation, a clinical picture further complicated by concurrent thrombocytopenia. Immunologically, serial biopsies confirmed the absence of HAR during this phase. Furthermore, rigorous viral surveillance testing for 47 designated pathogens, including PERV, PCMV, porcine lymphotropic herpesvirus-1 (PLHV1) and porcine reproductive and respiratory syndrome virus (PRRSV), remained negative [100].

#### 4.3.4. Emergence of Complications and Graft Explantation

Despite the initial clinical success, the biological limitations of the current genetic modifications eventually manifested. By postoperative day 31, the recipient developed progressive symptoms of xTMA. This was characterized by severe consumptive thrombocytopenia, microangiopathic hemolysis and surging levels of the terminal complement complex (sC5b-9). When targeted antibody therapies, including eculizumab and basiliximab, alongside plasma exchange, proved insufficient to halt the microvascular thrombosis, the team surgically explanted the auxiliary porcine liver on postoperative day 38 to prevent irreversible MOSF [100].

Following the explantation of the xenograft, the systemic xTMA was treated and successfully resolved using a combination of the terminal complement inhibitor eculizumab and therapeutic plasma exchange. Having survived the xenotransplantation and the subsequent xTMA crisis, the patient was maintained entirely by the hypertrophied regeneration of his native left lobe. Unfortunately, the underlying portal hypertension associated with his severe pre-existing cirrhosis eventually proved fatal. Beginning on postoperative day 135, the patient suffered massive, recurrent upper gastrointestinal hemorrhages secondary to gastric fundal varices. Despite aggressive interventions, including percutaneous transhepatic variceal embolization, the patient succumbed to recurrent hemorrhage on postoperative day 171 [100].

## 5. Comparative Analysis of the First Pig-to-Human Liver Xenotransplantations

To synthesize the distinct methodologies, divergent genetic architectures and highly varied clinical outcomes of these three trials, comprehensive comparative datasets are provided in Table 1, Table 2 and Table 3.

## 6. Current Limitations and Translational Challenges

Liver xenotransplantation faces substantial biological, physiological and translational barriers that must be acknowledged when interpreting the current evidence base. The following limitations define the boundaries of what can be concluded from the three first-in-human cases reviewed here and identify the principal challenges that must be resolved before broader clinical application can be considered.

### 6.1. Biological Limitations: Coagulation Dysregulation and Xenograft Thrombotic Microangiopathy

The most consistently observed and clinically limiting complication across the reported pig-to-human LxT cases was xTMA, characterized by consumptive thrombocytopenia, microangiopathic hemolysis and terminal complement activation [69,100]. Critically, xTMA emerged despite the use of current-generation 10-gene platforms specifically designed to address coagulation incompatibility, indicating that the existing suite of coagulation transgenes (hTBM, hEPCR, CD39) is insufficient to fully neutralize the pro-thrombotic environment created by porcine–human vascular contact. The molecular mechanisms driving xTMA are incompletely understood and likely multifactorial, involving residual incompatibilities in the complement–coagulation interface, aberrant platelet activation mediated by porcine vWF and inadequate macrophage inhibition despite CD47 expression [88,89,90,94]. Until xTMA can be reliably prevented, either through next-generation genetic engineering, perioperative pharmacological strategies or both, sustained xenograft function beyond a bridging interval cannot be assumed.

### 6.2. Remaining Immunological Barriers Beyond Hyperacute Rejection

While current TKO platforms have effectively eliminated HAR in the cases reviewed, this should not be interpreted as resolution of the immunological challenge. Acute humoral xenograft rejection, driven by de novo antibody formation against non-carbohydrate porcine antigens, and T-cell mediated cellular rejection remain active threats beyond the acute phase [52,60]. The immunosuppressive regimens employed in the Zhang et al. (2026) [100] case were intensive and multi-agent, carrying inherent risks of infectious complications and metabolic toxicity in an already compromised recipient. Furthermore, the two brain-dead decedent models used in the Tao et al. (2025) [98] and Shaked et al. (2026) [99] studies, while scientifically informative, cannot fully replicate the dynamic immunological responses of a living, immunocompetent recipient over weeks to months. The long-term immunological trajectory of pig-to-human liver xenotransplantation therefore remains substantially unknown.

### 6.3. Physiological Incompatibilities: Metabolic and Synthetic Mismatch

Beyond immunological rejection, the liver’s unique role as the primary metabolic and synthetic organ of the body creates incompatibilities that are not fully addressable through genetic engineering alone. The marked interspecies disparity in coagulation factor production observed in the Zhang et al. (2026) case, with porcine Factor VIII rising to 340-fold above the recipient’s baseline, illustrates that even functional xenograft activity can create pathological systemic consequences [100]. Similarly, species-specific differences in drug metabolism, lipoprotein synthesis, bile acid composition and hormonal regulation represent physiological mismatches that have not been systematically characterized in the human context. The current evidence base does not allow conclusions to be drawn about the long-term metabolic consequences of sustained exposure to a porcine hepatic synthetic profile in a human recipient.

### 6.4. Translational Challenges: Reproducibility, Scalability and Regulatory Barriers

The three cases reviewed represent heterogeneous procedures conducted across different institutions, employing distinct donor pig genotypes, surgical configurations and immunosuppressive protocols, making direct comparison difficult and generalizable conclusions premature. None of the studies were designed as controlled clinical trials. Two were conducted in brain-dead decedents and one under compassionate use authorization, settings that, while scientifically necessary as first steps, are not equivalent to prospective clinical trial conditions. Scalability also remains unproven; the production of genetically engineered donor pigs of consistent quality, appropriate size and confirmed pathogen-free status at the volumes required for even a modest clinical program presents significant logistical and regulatory challenges [101]. Regulatory frameworks governing first-in-human xenotransplantation trials are still nascent in most jurisdictions, and the absence of standardized endpoints, follow-up protocols and biosafety monitoring requirements across centers risks producing a fragmented evidence base that is difficult to meta-analyze or use for regulatory submissions.

### 6.5. Scientific Uncertainty and Ongoing Controversies

Several fundamental questions in the field remain unresolved and are the subject of active scientific debate. The relative contribution of complement activation versus coagulation dysregulation to xTMA pathogenesis is not yet established [94,95], with implications for whether future prevention strategies should prioritize genomic or pharmacological approaches. The clinical relevance of PERV and other porcine zoonotic pathogens over long follow-up periods, while reassuring in the short-term data reviewed here, cannot be excluded on the basis of weeks of observation [101,102]. Whether the auxiliary configuration used in the Zhang et al. (2026) [100] case offers meaningful immunological advantages over orthotopic replacement, or whether the survival of the native liver confounds the interpretation of xenograft function, remains an open question. Finally, the appropriate patient population for xenogeneic liver bridging, whether ALF, ACLF, or selected oncological indications as in the Zhang et al. case, has not been defined, and the risk–benefit calculus will differ substantially across these groups.

### 6.6. Economic Implications and Health Equity

The economic and health equity dimensions of clinical LxT demand parallel consideration alongside its biological advances. Conventional liver transplantation already constitutes one of the most economically burdensome surgical interventions, with total inpatient hospitalization costs in the United States rising from $945 million to $1.14 billion between 2016 and 2019 [103]. The cost infrastructure required for porcine LxT is expected to substantially exceed this baseline, encompassing biosecure breeding and pathogen-free certification of genetically engineered donor pigs, multi-agent immunosuppressive protocols incorporating rituximab, eculizumab and targeted biologics, intensive perioperative coagulation management and lifelong multiviral biosurveillance, none of which have yet been subjected to formal health technology assessment. This economic burden is particularly concerning, given the well-documented structural inequities that already characterize access to conventional liver transplantation: non-Hispanic Black patients are underrepresented on transplant waiting lists at over 80% of US transplant centers, with insurance status and socioeconomic position identified as the dominant structural predictors of listing inequity, disparities that persist despite iterative allocation policy reforms [104,105,106].

Introducing a novel, high-cost intervention concentrated in a small number of specialized academic centers risks amplifying these pre-existing inequities: Johnson has explicitly warned that xenotransplantation amplifies existing justice concerns in organ transplantation and could worsen them if equitable access frameworks are not embedded into its regulatory architecture from the outset [107], a concern independently articulated across the bioethical literature [47,108,109].

Patient selection criteria for xenotransplant trials present a related tension: current recommendations restricting eligibility to seriously ill patients for whom no alternatives exist, while prudent from a risk–benefit standpoint, have been criticized for conflicting with the principles of equitable subject selection and for potentially compromising the integrity of informed consent [110]. Paradoxically, if porcine bridging therapies can be optimized and manufactured at scale, they hold theoretical potential to extend hepatic support beyond the structural ceiling imposed by the global shortage of human allografts, a ceiling that, as discussed in Section 1.1, disproportionately burdens lower-income health systems where transplantation coverage can be as low as 16% of listed demand [11], but realizing this equitable potential will require deliberate investment in regulatory standardization, health technology assessment and access policy frameworks from the outset, rather than as a belated corrective to a technology already stratified along socioeconomic lines [109].

### 6.7. The Gap Between Experimental Success and Clinical Applicability

It is important to acknowledge that the distance between a proof-of-concept demonstration and a safe, reproducible, ethically governed clinical therapy is substantial. The cases reviewed here establish that porcine LxT is biologically possible in the human setting; they do not establish that it is ready for broader clinical deployment. Structured multicentre trials with prospectively defined endpoints, standardized donor platforms and rigorous long-term biosafety surveillance will be required before xenogeneic liver bridging can be considered a validated therapeutic option. The field is at an early and critical point, and intellectual honesty about the gap between current experimental findings and clinical applicability is essential to maintaining scientific credibility and appropriate patient expectations.

### 6.8. Summary and Comparative Synthesis of Liver Xenotransplantation Eras

The historical trajectory of LxT is defined by two distinct stages: the early search for phylogenetic proximity in NHP, and the modern era of precision porcine genome engineering. While the shift to pigs has successfully overcome HAR, the emergence of xTMA represents the new frontier of species incompatibility. A comprehensive side-by-side comparison of these donor species, genetic strategies, and clinical outcomes is synthesized in Table 4.

## 7. Future Perspectives and Conclusions

Bioartificial liver systems have been developed as BTs for ALF, but their clinical implementation remains limited by engineering constraints such as diffusion limitations in hollow-fiber bioreactor designs and challenges in maintaining sufficient functional hepatocyte mass within these systems [111,112,113,114]. In addition, the procurement and maintenance of functional hepatocytes pose logistical challenges because primary hepatocytes rapidly lose functional phenotype ex vivo and suitable donor tissue remains limited [115,116,117,118]. Direct hepatocyte transplantation, in which isolated hepatocytes are delivered into the portal circulation or spleen, represents a less invasive approach to temporary hepatic support that can partially restore synthetic and detoxification function while awaiting native liver regeneration or allotransplantation [119].

Although pioneering clinical work demonstrated proof-of-concept for this strategy, its impact in isolation has remained modest, largely because of the limitations shared with bioartificial systems [119]. Human-induced pluripotent stem cells (iPSC) have emerged as a theoretically unlimited and potentially autologous alternative. iPSC-derived hepatocyte-like cells have improved survival in experimental ALF models with detectable in vivo albumin production, and combining iPSC-derived hepatocytes with endothelial cells has been shown to prolong functional cell engraftment after transplantation [120].

At the organ-engineering level, decellularization of whole porcine livers yields extracellular matrix scaffolds that preserve the native three-dimensional hepatic microenvironment and can be recellularized with iPSC-derived hepatocytes or other cell sources, with bioengineered liver constructs containing porcine hepatocytes and endothelial cells demonstrating function after transplantation in preclinical liver failure models [121,122,123]. Nevertheless, whole-organ recellularization and 3D-bioprinted liver constructs remain preclinical and require substantial optimization before human use [121,122].

These converging regenerative technologies identify a plausible longer-term trajectory in which genetically modified porcine livers serve as the near-term clinical bridge while iPSC-derived constructs and bioengineered scaffolds mature toward autologous or immune-tolerant liver replacement; the decellularized porcine liver scaffold itself represents a point of direct intersection between xenogeneic and regenerative strategies, as the pig provides not only a transplantable organ but potentially also the biomaterial platform for future human liver bioengineering [121,122,123].

Conversely, genetically engineered pigs represent a potentially scalable source of transplantable organs because of their high reproductive capacity, short maturation time and physiologic similarities with humans [65,124,125]. Gene-edited pigs may therefore help address the persistent shortage of donor organs in transplantation medicine and recent experimental work has explored the use of porcine livers in extracorporeal perfusion circuits, demonstrating that genetically modified pig livers can be maintained in extracorporeal systems and retain functional parameters such as bile production and metabolic activity over extended perfusion periods [99,126,127].

The three pig-to-human LxT procedures described in this review collectively suggest that genetically modified porcine livers may be capable of providing hepatic synthetic, metabolic and detoxification support within the human circulatory environment, representing an important proof-of-concept advance. Crucially, however, the totality of current evidence supports a specific and circumscribed clinical role for xenogeneic liver support: that of a temporary bridging strategy, maintaining physiological viability until native liver recovery or human allotransplantation becomes possible, rather than a definitive organ replacement. The consistent emergence of xTMA across cases appears to represent a principal remaining barrier to durable graft function [69,100], and the path toward any more extended clinical application will require both refined genetic engineering strategies and coordinated pharmacological adjuncts.

The prevention of xTMA is likely to require a multi-pronged approach. Evidence from pig-to-human kidney xenotransplantation has demonstrated that terminal complement inhibition with eculizumab, when initiated preoperatively, can prevent the development of xTMA [128]. In the context of LxT, the massive sC5b-9 surge documented on postoperative day 27 in the Zhang et al. (2026) case suggests that sustained complement dysregulation is a primary pathophysiological driver [100]. At the genomic level, future donor pig engineering should incorporate transgenes targeting proximal complement convertases alongside humanization of porcine vWF, which has been shown to reduce aberrant platelet activation and sequestration in xenotransplantation models [99]. Additionally, targeted deletion of the asialoglycoprotein receptor 1, responsible for hepatic phagocytic clearance of primate platelets, represents a further rational modification to reduce consumptive thrombocytopenia [90].

Beyond surgical implantation, the ELC model pioneered by Shaked et al. (2026) [99] provides a less invasive, rapidly reversible alternative for patients with fulminant ALF who are too hemodynamically unstable to undergo laparotomy. The integration of normothermic machine perfusion technology, already clinically approved for allograft preservation, with increasingly sophisticated genetically modified porcine livers represents a scalable and technically feasible paradigm for near-term clinical translation [129]. A stepwise strategy, in which extracorporeal xenogeneic liver support first stabilizes the patient metabolically and hemodynamically before definitive transplantation is undertaken, may constitute the most pragmatic near-term clinical pathway, particularly for ALF and ACLF presentations where critical decisions must be made under acute time pressure [71]. This is consistent with the currently available evidence, which supports xenogeneic liver support as a bridging modality rather than a standalone curative intervention.

The biosafety landscape for clinical xenotransplantation must evolve in parallel with its surgical and genetic advancements. Although PERV transmission to human recipients has not been documented in any of the three studies reviewed, longitudinal surveillance using metagenomic sequencing remains indispensable for the detection of unknown porcine pathogens and for the characterization of host–graft–microbiome interactions [101,102]. The International Xenotransplantation Association has issued consensus recommendations emphasizing the necessity of standardized biosecure breeding programs and recipient monitoring frameworks as prerequisites for safe multicenter expansion [101]. Furthermore, ethical frameworks governing patient selection criteria, informed consent for experimental surgical interventions and equitable access to these emerging therapies must be proactively developed as the field transitions from compassionate use toward structured clinical trials [130].

The first-in-human cases detailed in this review collectively represent the opening, not the conclusion, of a new chapter in transplantation medicine: one in which xenogeneic liver support may ultimately serve as a critical bridge within a broader, integrated transplantation strategy.

## Figures and Tables

**Table 1:** Comparative analysis of study design, donor genetics and surgical methodology.

Parameter	Tao et al. [98]	Shaked et al. [99]	Zhang et al. [100]
Recipient Profile	Brain-dead human decedent	Four brain-dead human decedents	Living human (71-year-old male)
Clinical Rationale	Preclinical feasibility/Bridging proof-of-concept	ALF/Acute-on-chronic liver failure (ACLF) extracorporeal bridging	Unresectable HCC, high rupture risk
Donor Breed	Bama miniature pig	Yucatan miniature pig	Diannan miniature pig (32 kg)
Total Gene Edits	6-Gene Platform	10-Gene (EGEN-5784) Platform	10-Gene Platform
Gene Knockouts (KO)	GGTA1, CMAH, β4GalNT2	GGTA1, CMAH, β4GalNT2, PERV pol	GGTA1, CMAH, β4GalNT2
Human Transgenes (KI)	CD46, CD55, hTBM	hEPCR, hTBM, TNFAIP3, HMOX1, CD46, CD55, CD47	CD46, CD55, CD59, CD39, hTBM, hEPCR, CD47
Surgical Strategy	Heterotopic auxiliary transplantation	Extracorporeal Liver Cross-circulation (ELC)	Orthotopic auxiliary transplantation
Vascular Anatomy	Portal vein to distal IVC; Hepatic artery to aorta; suprahepatic IVC to proximal IVC	Venous cannulation to isolated metra perfusion circuit	Native hepatic artery and portal vein anastomoses directly in right hepatic fossa
Native Liver Status	Retained in situ	Intact (Decedents 1–3); Total Hepatectomy (Decedent 4)	Left lobe retained; right lobe totally resected

**Table 2:** Comparative analysis of clinical outcomes, hepatic function and Zoonotic Surveillance.

Parameter	Tao et al. [98]	Shaked et al. [99]	Zhang et al. [100]
Graft Survival Duration	10 days (Electively terminated)	72–84 h (Decedents 1–3); 48 h anhepatic (Decedent 4)	38 days (Graft explanted due to xTMA)
Total Recipient Survival	10 days (Brain-dead model)	100 h max (Brain-dead model)	171 days (Living recipient)
Biliary Output	66.5 mL cumulative by day 10	19.4 mL/h average across Decedents 1–3; Output visibly reduced in Decedent 4 due to excessive metabolic demand post-hepatectomy	Peaked at 400 mL/day, stabilizing at 150 mL/day post-rituximab pulse
Hepatic Enzymes	Normal ALT; Transient massive AST spike on Day 1	Transient mild elevation in transaminases and bilirubin upon ELC start	Functional normalization; Temporary rise corrected on Day 8
Metabolic Stabilization	Porcine albumin robustly detected in serum	Maintained normal pH (7.35), lactate (2.3 mmol/L) and ammonia (50 µmol/L)	Rapid synthesis of porcine primary bile acids and porcine albumin
Zoonosis Surveillance	PERV and PCMV negative/diminished	Not explicitly detailed	Negative for PERV, PCV3, PCMV, PLHV, PRRSV-1/2
Microchimerism	Undetectable in recipient blood/tissue	Not explicitly detailed	Not explicitly detailed

**Table 3:** Comparative Analysis of Immunological, Hematological and Histopathological Metrics.

Parameter	Tao et al. [98]	Shaked et al. [99]	Zhang et al. [100]
Coagulation Profile	Transient thrombocytopenia; aPTT prolonged then recovered	Maintained normal INR (1.1) during 48 h anhepatic support	Early coagulopathy managed; Massive porcine FVIII output; Delayed xTMA
Immunosuppression	Anti-thymocyte globulin, eculizumab, etanercept, rituximab, FK506, MMF, methylprednisolone	Minimal (Intravenous methylprednisolone)	Basiliximab, rituximab, methylprednisolone, FK506, sirolimus, MMF
Cellular Infiltration	Minimal inflammatory cells; High Ki67 & CD31 repopulation	CD14+ macrophages/monocytes predominant; mild CD3+ T-cells	Moderate lymphocytic infiltration; No acute cellular rejection
Humoral/Complement	Low C3d/C4d/C5b-9 deposition; no significant serum IgG/IgM shifts	IgM deposition on endothelium; focal portal venule endotheliitis	Weak C4d initially; massive sC5b-9 spike (353 ng/mL) and CH50 spike by Day 27
Vascular Pathology	Intact LSEC fenestrae confirmed by SEM	Mild red blood cells extravasation expanding by 72 h	Fibrinous microthrombi and massive vWF staining characteristic of xTMA

**Table 4:** Comparative analysis of NHP-to-human and pig-to-human liver xenotransplantation.

Parameter	NHP-to-Human LxT	Pig-to-Human LxT
Donor Species	Chimpanzees (Pan troglodytes), 1966–1974; Baboons (*Papio* sp.), 1992–1993 [35]	Genetically engineered Sus scrofa domesticus: Bama, Yucatan and Diannan miniature pig breeds [98,99,100]
Total Human Clinical Cases	5 cases: 3 chimpanzee-to-human [35] 2 baboon-to-human [35,36,37]	3 cases (2025–2026): heterotopic auxiliary in brain-dead decedent (Tao et al.); extracorporeal liver cross-circulation in brain-dead decedents (Shaked et al.); orthotopic auxiliary in living recipient (Zhang et al.) [98,99,100]
Genetic Modification of Donor	None; wild-type donors throughout. Immunological tolerance relied entirely on phylogenetic proximity to Homo sapiens [35]	6–10 gene modifications via CRISPR/Cas9 and PiggyBac: triple-knockout (GGTA1, CMAH, β4GalNT2) combined with up to 7 human transgenes (CD46, CD55, CD59, hTBM, hEPCR, CD39, CD47); PERV pol inactivation in EGEN-5784 [66,98,99,100]
Hyperacute Rejection	Low–moderate risk due to phylogenetic proximity; complement activation via C3/C5 and the membrane attack complex still contributed to graft injury [36,39,40,41]	High risk in wild-type pigs; preformed anti-α-Gal antibodies (1–4% of total Ig) activate complement within seconds. Effectively abolished in current TKO platforms; no HAR observed in any of the three human cases [52,58,66,98,99,100]
Principal Immunological and Biological Barrier	Complement-mediated humoral rejection uncontrolled by FK506; biliary and hepatobiliary physiological incompatibility [36,39,40,41,42]	xTMA: consumptive thrombocytopenia microangiopathic hemolysis terminal complement activation emerging despite 10-gene platforms [69,100]
Coagulation Profile	Moderate mismatch synthetic function confirmed with shift of serum albumin and clotting factors to baboon isoforms in Case 1 biliary sludge was the dominant hepatobiliary finding at autopsy [36,42]	Severe dysregulation: consumptive thrombocytopenia driven by porcine vWF–platelet GPIbα mismatch Kupffer cellphagocytosis and xTMA porcine Factor VIII rose to 340-fold above baseline in the living recipient [88,89,90,94,95,100]
Biliary Function	Entire intrahepatic biliary tree occluded by biliary sludge at autopsy in Case 1 despite patent choledochojejunostomy; identified as a principal cause of graft dysfunction [36,42]	Confirmed in all three cases: 66.5 mL cumulative at day 10 (Tao et al.); 19.4 mL/h average (Shaked et al.); peaked at 400 mL/day stabilizing at 150 mL/day (Zhang et al.) [98,99,100]
Metabolic and Synthetic Function	Demonstrated in baboon Case 1: progressive shift of serum proteins to baboon isoforms confirmed active hepatic synthesis, rendering the patient a functional chimera [36]	Confirmed across cases: porcine albumin detected in serum; normal pH 7.35, lactate 2.3 mmol/L, ammonia 50 μmol/L and INR 1.1 maintained during 48 h anhepatic phase (Shaked); porcine bile acids and coagulation factors synthesized (Zhang) [98,99,100]
Longest Reported Survival and Cause of Death	70 days (baboon Case 1, 1992): death from subarachnoid hemorrhage due to invasive aspergillosis; viable graft with minimal rejection confirmed at autopsy [36] 26 days (baboon Case 2, 1993): death from peritonitis secondary to anastomotic leak [37]	171 days (living recipient, Zhang et al. 2026 [100]): graft explanted on day 38 due to progressive xTMA; patient survived on regenerated native left lobe, dying from recurrent gastric fundal variceal hemorrhage secondary to pre-existing portal hypertension [100]
Immunosuppression Strategy	Chimpanzee era: azathioprine, corticosteroids, ALG [34]. Baboon era: FK506, prednisone, prostaglandin, cyclophosphamide; T-cell rejection suppressed but innate/humoral responses uncontrolled [36,37]	Multi-agent induction and maintenance: rituximab, basiliximab, methylprednisolone, FK506, sirolimus, MMF; eculizumab for complement inhibition; ATG and etanercept in the Tao et al. protocol [98,99,100]
Zoonotic and Biosafety Risk	High: simian foamy virus and simian immunodeficiency virus are evolutionarily ready to jump to humans; cross-species simian foamy virus transmission to humans documented [43,44]. Prompted joint FDA/US Public Health Service guidelines halting clinical primate xenotransplantation [48]	Lower: PERV not transmitted in any reported human case; PCMV, PLHV1, PRRSV and PCV3 undetected throughout. Designated pathogen-free breeding and metagenomic surveillance mandated by the International Xenotransplantation Association [98,100,101,102]
Donor Availability and Scalability	Extremely limited: cannot be farmed; ethical constraints on primate use; colony sizes inadequate for any clinical program [45,46]	Potentially unlimited: pigs breed rapidly, reach maturity quickly and are already used in food production. Scalable biosecure GE pig production technically feasible, but not yet standardized for clinical-grade donors [46,65]
Ethical and Regulatory Status	Highly controversial: use of cognitively complex primates raised acute animal welfare and societal concerns alongside zoonosis risks; clinical NHP xenotransplantation halted by regulatory guidelines [45,46,47,48]	More societally acceptable given established use as food animals. Compassionate use approvals obtained for all three human cases. Equity and access concerns identified; regulatory frameworks and International Xenotransplantation Association biosafety guidelines are nascent [47,100,101,107,108,109,110]

## Data Availability

The data presented in this review are derived from previously published studies, which are cited throughout the text and listed in the references. No new primary data were created.

## Abbreviations

The following abbreviations are used in this manuscript:

LT	Liver Transplantation
ALF	Acute Liver Failure
WL	Waiting List
BTs	Bridging Therapies
MOSF	Multiorgan System Failure
INR	International Normalized Ratio
DAMPs	Damage-Associated Molecular Patterns
CRRT	Continuous Renal Replacement Therapy
LxT	Liver Xenotransplantation
NHP	Non-Human Primate
FK506	Tacrolimus
HBV	Hepatitis B Virus
HAR	Hyperacute Rejection
GGTA1	α-1,3-galactosyltransferase gene
GTKO	α-1,3-galactosyltransferase gene-knockout
Neu5Gc	N-glycolylneuraminic acid
CMAH	Cytidine monophospho-N-acetylneuraminic acid hydroxylase
β4GalNT2	β-1,4-N-acetylgalactosaminyl transferase 2
Sd(a)	DBA-reactive glycans
TKO	Triple-Knockout
hTBM	Human Thrombomodulin
hEPCR	Human Endothelial Protein C Receptor
vWF	Von Willebrand Factor
xTMA	Xenograft thrombotic microangiopathy
IVC	Inferior Vena Cava
ALT	Alanine aminotransferase
AST	Aspartate aminotransferase
aPTT	Activated partial thromboplastin time
LSEC	Liver sinusoidal endothelial cell
SEM	Scanning electron microscopy
PERV	Porcine endogenous retrovirus
PCMV	Porcine cytomegalovirus
ELC	Extracorporeal Liver Cross-Circulation
TNFAIP3	Tumor necrosis factor alpha-induced protein 3
HMOX1	Heme Oxygenase 1
HCC	Hepatocellular carcinoma
MMF	Mycophenolate mofetil
PLHV1	Porcine lymphotropic herpesvirus-1
PRRSV	Porcine reproductive and respiratory syndrome virus
ACLF	Acute-on-chronic liver failure
PCV3	Porcine circovirus type 3
iPSC	Induced pluripotent stem cells
